# Supplementary material for: Current Status of Putative Animal Sources of SARS-CoV-2 Infection in Humans: Wildlife, Domestic Animals and Pets
Source: Microorganisms. 2021 Apr 17;9(4):868. doi: 10.3390/microorganisms9040868 (PMC8072559; doi:10.3390/microorganisms9040868)
Supplement: Supplementary file 1 [file microorganisms-09-00868-s001.zip › microorganisms-1168666-supplementary.pdf]

Supplementary Table S1. SARS-CoV-2 genomes from animal strains, according to GISAID (gisaid.org).

Animal Host	Virus Name	Accession ID	Location
Canis lupus familiaris	hCoV-19/dog/Hong Kong/20-03695/2020	EPI_ISL_450403	Asia/Hong Kong
	hCoV-19/dog/USA/TX-TAMU-077/2020	EPI_ISL_699508	North America/USA/Texas/Brazos County
	hCoV-19/dog/Netherlands/un-EMC-2/2020	EPI_ISL_722380	Europe/Netherlands
	hCoV-19/dog/Italy/Dog399-20BA/2020	EPI_ISL_730652	Europe/Italy/Apulia
	hCoV-19/dog/Brazil/RJ-FIOCRUZ-A004N/2020	EPI_ISL_848072	South America/Brazil/Rio de Janeiro
	hCoV-19/dog/Brazil/RJ-FIOCRUZ-A118/2020	EPI_ISL_848073	South America/Brazil/Rio de Janeiro
	hCoV-19/dog/Brazil/RJ-FIOCRUZ-A119/2020	EPI_ISL_848074	South America/Brazil/Rio de Janeiro
	hCoV-19/dog/Brazil/RJ-FIOCRUZ-A136/2020	EPI_ISL_848075	South America/Brazil/Rio de Janeiro
	hCoV-19/dog/Brazil/RJ-FIOCRUZ-A016NS3/2020	EPI_ISL_848076	South America/Brazil/Rio de Janeiro
	hCoV-19/dog/Brazil/RJ-FIOCRUZ-A301/2020	EPI_ISL_848077	South America/Brazil/Rio de Janeiro
	hCoV-19/dog/Brazil/RJ-FIOCRUZ-A398/2020	EPI_ISL_848078	South America/Brazil/Rio de Janeiro
	hCoV-19/dog/Brazil/RJ-FIOCRUZ-A399/2020	EPI_ISL_848079	South America/Brazil/Rio de Janeiro
	hCoV-19/dog/Brazil/RJ-FIOCRUZ-A401/2020	EPI_ISL_848080	South America/Brazil/Rio de Janeiro
	hCoV-19/dog/Brazil/RJ-FIOCRUZ-A403/2020	EPI_ISL_848081	South America/Brazil/Rio de Janeiro
	hCoV-19/dog/Brazil/RJ-FIOCRUZ-A404/2020	EPI_ISL_848082	South America/Brazil/Rio de Janeiro
	hCoV-19/dog/Portugal/Dog42/2021	EPI_ISL_1220542	Europe/Portugal/Coimbra
Felis catus	hCoV-19/cat/USA/FL-BPHL-1083/2021	EPI_ISL_1218884	North America/USA/Florida
	hCoV-19/cat/USA/FL-BPHL-1082/2021	EPI_ISL_1218883	North America/USA/Florida
	hCoV-19/cat/USA/FL-BPHL-1081/2021	EPI_ISL_1218882	North America/USA/Florida
	hCoV-19/cat/Switzerland/ZH-UZH-Cat1/2020	EPI_ISL_1005699	Europe/Switzerland/Zurich
	hCoV-19/cat/Italy/VE-IZSVe-20DIA30040-2/2020	EPI_ISL_962892	Europe/Italy/Veneto/Venezia
	hCoV-19/cat/France/un-ENVT-LHOOQ/2020	EPI_ISL_873210	Europe/France
	hCoV-19/cat/Russia/LEN-RII-22390S/2021	EPI_ISL_873040	Europe/Russia/Leningrad/Vsevolozhsk
	hCoV-19/cat/Brazil/RJ-FIOCRUZ-A408/2020	EPI_ISL_848083	South America/Brazil/Rio de Janeiro
	hCoV-19/cat/Brazil/RJ-FIOCRUZ-A003N/2020	EPI_ISL_848071	South America/Brazil/Rio de Janeiro
	hCoV-19/cat/Brazil/RJ-FIOCRUZ-A002N/2020	EPI_ISL_848070	South America/Brazil/Rio de Janeiro
	hCoV-19/cat/Russia/RII-LEN-22246S/2021	EPI_ISL_811147	Europe/Russia/Leningrad/Vsevolozhsk
	hCoV-19/cat/Iran/MeshkinShahr/2020	EPI_ISL_803107	Asia/Iran
	hCoV-19/cat/Hong Kong/20-04236/2020	EPI_ISL_759858	Asia/Hong Kong
	hCoV-19/cat/Netherlands/UN-EMC-1/2020	EPI_ISL_722300	Europe/Netherlands
	hCoV-19/cat/Greece/2K/2020	EPI_ISL_717979	Europe/Greece/Thessaloniki
	hCoV-19/cat/USA/TX-TAMU-078/2020	EPI_ISL_699509	North America/USA/Texas/Brazos County
	hCoV-19/cat/USA/TX-TAMU-057/2020	EPI_ISL_699507	North America/USA/Texas/Brazos County
	hCoV-19/cat/USA/TX-TAMU-013/2020	EPI_ISL_699506	North America/USA/Texas/Brazos County
	hCoV-19/cat/Denmark/mDK-317/2020	EPI_ISL_683166	Europe/Denmark
	hCoV-19/cat/Denmark/mDK-316/2020	EPI_ISL_683165	Europe/Denmark
	hCoV-19/cat/Denmark/mDK-315/2020	EPI_ISL_683164	Europe/Denmark
	hCoV-19/cat/England/CVR-Cat2/2020	EPI_ISL_536400	Europe/United Kingdom/England
	hCoV-19/cat/Belgium/BE-MG-0320/2020	EPI_ISL_487275	Europe/Belgium
	hCoV-19/cat/France/Env-Di/2020	EPI_ISL_483064	Europe/France
	hCoV-19/cat/France/Env-Ba/2020	EPI_ISL_483063	Europe/France
	hCoV-19/cat/Spain/CT-IrsiCaixa-G5/2020	EPI_ISL_482820	Europe/Spain/Catalunya/Barcelona
	hCoV-19/cat/USA/NYAD2/2020	EPI_ISL_450407	North America/USA
	hCoV-19/cat/USA/NYAD1/2020	EPI_ISL_450406	North America/USA
	hCoV-19/cat/France/IDF-53/2020	EPI_ISL_437349	Europe/France/Île-de-France/Essone
	hCoV-19/cat/China/Wuhan/2020	EPI_ISL_421531	Asia/China/Wuhan
Gorilla gorilla gorilla	hCoV-19/gorilla/USA/CA-ALSR-5742/2021	EPI_ISL_862722	North America/USA/California/San Diego
Chlorocebus sabaeus	hCoV-19/monkey/USA/MD-USAMRIID-AGM/2020	EPI_ISL_825033	North America/USA/Maryland
Manis javanica	hCoV-19/pangolin/Guangdong/SM79-9/2019	EPI_ISL_471470	Asia/China/Guangdong
	hCoV-19/pangolin/Guangdong/SM44-9/2019	EPI_ISL_471469	Asia/China/Guangdong
	hCoV-19/pangolin/Guangdong/FM45-9/2019	EPI_ISL_471468	Asia/China/Guangdong
	hCoV-19/pangolin/Guangdong/A22-2/2019	EPI_ISL_471467	Asia/China/Guangdong
	hCoV-19/pangolin/Guangdong/cDNA31-S/2019	EPI_ISL_471466	Asia/China/Guangdong
	hCoV-19/pangolin/Guangdong/cDNA20-S/2019	EPI_ISL_471465	Asia/China/Guangdong
	hCoV-19/pangolin/Guangdong/cDNA18-S/2019	EPI_ISL_471464	Asia/China/Guangdong

	hCoV-19/pangolin/Guangdong/cDNA16-S/2019	EPI_ISL_471463	Asia/China/Guangdong
	hCoV-19/pangolin/Guangdong/cDNA9-S/2019	EPI_ISL_471462	Asia/China/Guangdong
	hCoV-19/pangolin/Guangdong/cDNA8-S/2019	EPI_ISL_471461	Asia/China/Guangdong
	hCoV-19/pangolin/China/MP789/2019	EPI_ISL_412860	Asia/China
	hCoV-19/pangolin/Guangdong/1/2019	EPI_ISL_410721	Asia/China/Guangdong
	hCoV-19/pangolin/Guangdong/P2S/2019	EPI_ISL_410544	Asia/China/Guangdong
	hCoV-19/pangolin/Guangxi/P3B/2017	EPI_ISL_410543	Asia/China/Guangxi
	hCoV-19/pangolin/Guangxi/P2V/2017	EPI_ISL_410542	Asia/China/Guangxi
	hCoV-19/pangolin/Guangxi/P5E/2017	EPI_ISL_410541	Asia/China/Guangxi
	hCoV-19/pangolin/Guangxi/P5L/2017	EPI_ISL_410540	Asia/China/Guangxi
	hCoV-19/pangolin/Guangxi/P1E/2017	EPI_ISL_410539	Asia/China/Guangxi
	hCoV-19/pangolin/Guangxi/P4L/2017	EPI_ISL_410538	Asia/China/Guangxi
Manis pentadactyla	hCoV-19/pangolin/Yunnan/MP20/2017	EPI_ISL_610156	Asia/China/Yunnan
Mus musculus	hCoV-19/mouse/Wuhan/LG/2020	EPI_ISL_1039212	Asia/China/Wuhan (BALB/c mouse)
	hCoV-19/mouse/Harbin/HRB-26m/2020	EPI_ISL_459910	Asia/China/Heilongjiang/Harbin
Mustela lutreola	hCoV-19/mink/Netherlands/NB04_index/2020	EPI_ISL_447634	Europe/Netherlands
	hCoV-19/mink/Netherlands/NB03_index/2020	EPI_ISL_447633	Europe/Netherlands
	hCoV-19/mink/Netherlands/NB02_16RS/2020	EPI_ISL_447632	Europe/Netherlands
	hCoV-19/mink/Netherlands/NB02_index/2020	EPI_ISL_447631	Europe/Netherlands
	hCoV-19/mink/Netherlands/NB02_13KS/2020	EPI_ISL_447630	Europe/Netherlands
	hCoV-19/mink/Netherlands/NB02_07KS/2020	EPI_ISL_447629	Europe/Netherlands
	hCoV-19/mink/Netherlands/NB02_06KS/2020	EPI_ISL_447628	Europe/Netherlands
	hCoV-19/mink/Netherlands/NB02_03KS/2020	EPI_ISL_447627	Europe/Netherlands
	hCoV-19/mink/Netherlands/NB01_04KS/2020	EPI_ISL_447626	Europe/Netherlands
	hCoV-19/mink/Netherlands/NB01_03KS/2020	EPI_ISL_447625	Europe/Netherlands
	hCoV-19/mink/Netherlands/NB01_02KS/2020	EPI_ISL_447624	Europe/Netherlands
	hCoV-19/mink/Netherlands/NB01_01KS/2020	EPI_ISL_447623	Europe/Netherlands
	hCoV-19/mink/Netherlands/1/2020	EPI_ISL_431778	Europe/Netherlands/Noord Brabant/Milheeze
Neovison vison	902 viruses, from North America (USA including Winsconsin and Michigan, and Canada) and Europe (the Netherlands, Denmark, Lithuania, and Poland)		
Panthera leo	hCoV-19/lion/USA/NY-2/2020	EPI_ISL_566044	North America/USA/New York
	hCoV-19/lion/USA/NY-041520/2020	EPI_ISL_566038	North America/USA/New York
	hCoV-19/lion/USA/NY-3-041520/2020	EPI_ISL_566037	North America/USA/New York
	hCoV-19/lion/USA/NY-2-041520/2020	EPI_ISL_566036	North America/USA/New York
Panthera tigris jacksoni	hCoV-19/tiger/USA/NY-P4/2020	EPI_ISL_566043	North America/USA/New York
	hCoV-19/tiger/USA/NY-4-040420/2020	EPI_ISL_566042	North America/USA/New York
	hCoV-19/tiger/USA/NY-3-040420/2020	EPI_ISL_566041	North America/USA/New York
	hCoV-19/tiger/USA/NY-P3/2020	EPI_ISL_566040	North America/USA/New York
	hCoV-19/tiger/USA/NY-2-040420/2020	EPI_ISL_566039	North America/USA/New York
	hCoV-19/tiger/USA/NY-040420/2020	EPI_ISL_420293	North America/USA/New York
Rhinolophus affinis	hCoV-19/bat/Yunnan/RaTG13/2013	EPI_ISL_402131	Asia/China/Yunnan/Puer City
Rhinolophus bat	hCoV-19/bat/Yunnan/PrC31/2018	EPI_ISL_1098866	Asia/China/Yunnan/Puer City

Supplementary Table S2. Reports of pets diagnosed with COVID-19 using SARS-CoV-2 RT-PCR

Date of Start	Area	Pets per House	Clinical Symptoms	Sources
**ASIA**				
**Hong Kong**				
02/26/20	Hong Kong	1 dog	Asymptomatic	Sit et al. Infection of dogs with SARS-CoV-2. Nature 2020; 586:776-8. https://www.oie.int/wahis_2/public/wahid.php/Reviewreport/Review?page_refer=MapFullEventReport&reportid=33455&newlang=fr
03/18/20	Hong Kong	1 dog	Asymptomatic	https://www.oie.int/wahis_2/public/wahid.php/Reviewreport/Review?page_refer=MapFullEventReport&reportid=33684&newlang=en
03/30/20	Hong Kong	1 cat	Asymptomatic	https://www.oie.int/wahis_2/public/wahid.php/Reviewreport/Review?page_refer=MapFullEventReport&reportid=33832&newlang=en
07/13/20	Hong Kong	1 cat	Asymptomatic	https://www.oie.int/wahis_2/public/wahid.php/Reviewreport/Review?reportid=35452
07/21/20	Hong Kong	1 cat	Asymptomatic	https://www.oie.int/wahis_2/public/wahid.php/Reviewreport/Review?page_refer=MapFullEventReport&reportid=35169
07/28/20	Hong Kong	1 cat	Asymptomatic	https://www.oie.int/wahis_2/public/wahid.php/Reviewreport/Review?reportid=35351
07/31/20	Hong Kong	1 dog	Asymptomatic	
07/31/20	Hong Kong	1 cat	Asymptomatic	
08/04/20	Hong Kong	1 cat	Asymptomatic	
08/06/20	Hong Kong	1 dog	Asymptomatic	
11/23/20	Hong Kong	1 dog	Asymptomatic	https://www.oie.int/wahis_2/public/wahid.php/Reviewreport/Review?page_refer=MapFullEventReport&reportid=36751
11/23/20	Hong Kong	1 dog	Asymptomatic	https://www.oie.int/wahis_2/public/wahid.php/Reviewreport/Review?reportid=37029
11/25/20	Hong Kong	1 cat	Asymptomatic	https://www.oie.int/wahis_2/public/wahid.php/Reviewreport/Review?page_refer=MapFullEventReport&reportid=36893
12/12/20	Hong Kong	1 dog	Asymptomatic	https://www.oie.int/wahis_2/public/wahid.php/Reviewreport/Review?page_refer=MapFullEventReport&reportid=37150
12/14/20	Hong Kong	1 dog	Asymptomatic	
12/15/20	Hong Kong	1 dog	Asymptomatic	
12/30/2020	Hong Kong	1 cat	Asymptomatic	https://www.oie.int/wahis_2/public/wahid.php/Reviewreport/Review?reportid=37454
**Japan**				
07/26/20	Tokyo	1 dog	Asymptomatic	https://www.oie.int/wahis_2/public/wahid.php/Reviewreport/Review?page_refer=MapFullEventReport&reportid=35864
07/31/20	Tokyo	1 dog	Asymptomatic	
08/07/20	Tokyo	1 dog	Asymptomatic	
08/12/20	Tokyo	1 dog	Asymptomatic	
09/12/20	Tokyo	2 cats	Asymptomatic	https://www.oie.int/wahis_2/public/wahid.php/Reviewreport/Review?reportid=36420
**EUROPE**				
03/18/20	Belgium	1 cat	Digestive and respiratory signs	Garigliany M, et al. SARS-CoV-2 Natural Transmission from Human to Cat, Belgium, March 2020. Emerg Infect Dis 2020; 26:3069-71
04/22/20	Spain (Catalonia)	1 cat	Respiratory signs	https://www.plateforme-esa.fr/article/covid-19-et-animaux-mise-a-jour-au-05-01-2021
05/02/20	France (Paris)	1 cat	Digestive and respiratory signs	Sailleau C, et al. First detection and genome sequencing of SARS-CoV-2 in an infected cat in France. Transbound Emerg Dis 2020; 67:2324-8.
05/11/20	Italy	1 dog	Asymptomatic	https://promedmail.org/promed-post/?id=7934864
05/12/20	France (Bordeaux)	1 cat	Digestive and respiratory signs	http://www.envt.fr/content/communiqu%C3%A
05/13/20	Germany (Palatinate, Bavaria)	1 cat	Asymptomatic	https://promedmail.org/promed-post/?id=7332909
05/18/20	Russia (Moscow area)	1 cat[1]	Asymptomatic	https://www.oie.int/wahis_2/public/wahid.php/Reviewreport/Review?page_refer=MapFullEventReport&reportid=34443
06/13/20	Denmark	1 dog[2]	Asymptomatic	https://promedmail.org/promed-post/?id=20200624.7506728

	(North Jutland)			
07/27/20	United Kingdom (South England)	1 cat	Respiratory signs	https://promedmail.org/promed-post/?id=7617582 https://www.oie.int/wahis_2/public/wahid.php/Reviewreport/Review?page_refer=MapFullEventReport&reportid=35182
12/01/20	Slovenia	1 ferret	Digestive signs	https://www.oie.int/wahis_2/public/wahid.php/Reviewreport/Review?page_refer=MapFullEventReport&reportid=37289
12/03/20	Switzerland (Zurich)	1 cat	Respiratory signs	https://www.oie.int/fileadmin/Home/MM/Switzerland_03.12.2020_Research_on_SARS-CoV-2_-_confirmation_in_a_cat_in_the_Canton_of_Zurich.pdf
**NORTH AMERICA**				
**United States of America**				
04/01/20	New-York	1 cat	Sneezing and eyes discharge	https://www.oie.int/wahis_2/public/wahid.php/Reviewreport/Review?reportid=34086
04/06/20	New-York	1 cat[3]	Sneezing and eyes discharge	https://www.cdc.gov/media/releases/2020/s0422-covid-19-cats-NYC.html
04/15/20	New-York	1 dog	Respiratory signs	https://www.oie.int/wahis_2/public/wahid.php/Reviewreport/Review?reportid=34525
05/19/20	Illinois (Cook)	1 cat[1]	Respiratory signs	https://www.oie.int/wahis_2/public/wahid.php/Reviewreport/Review?reportid=34590 https://www.aphis.usda.gov/aphis/ourfocus/animalhealth/sa_one_health/sars-cov-2-animals-us
05/20/20	Minnesota (Carver)	1 cat	Respiratory signs	https://www.oie.int/wahis_2/public/wahid.php/Reviewreport/Review?page_refer=MapFullEventReport&reportid=34548
06/22/20	Georgia (Berrien)	1 dog	Neurological signs[4]	https://www.oie.int/wahis_2/public/wahid.php/Reviewreport/Review?reportid=34937
06/25/20	California (Orange)	1 cat	Asymptomatic	https://www.oie.int/wahis_2/public/wahid.php/Reviewreport/Review?reportid=34991 https://www.aphis.usda.gov/aphis/ourfocus/animalhealth/sa_one_health/sars-cov-2-animals-us
06/26/20	Texas (Tarrant)	1 dog	Asymptomatic	https://www.oie.int/wahis_2/public/wahid.php/Reviewreport/Review?reportid=34991 https://promedmail.org/promed-post/?id=7554832
06/26/20	South Carolina (Charleston)	1 dog	Asymptomatic	https://www.oie.int/wahis_2/public/wahid.php/Reviewreport/Review?reportid=35051 https://promedmail.org/promed-post/?id=7588843
06/28/20	Texas (Brazos)	1 cat	Asymptomatic	https://www.oie.int/wahis_2/public/wahid.php/Reviewreport/Review?page_refer=MapFullEventReport&reportid=35140
07/10/20	Arizona (Maricopa)	1 dog	Respiratory signs	https://www.oie.int/wahis_2/public/wahid.php/Reviewreport/Review?page_refer=MapFullEventReport&reportid=35140
07/14/20	Georgia (Coweta)	1 cat	Respiratory signs	https://www.oie.int/wahis_2/public/wahid.php/Reviewreport/Review?reportid=35525
07/17/20	Texas (Brazos)	1 cat	Asymptomatic	https://www.oie.int/wahis_2/public/wahid.php/Reviewreport/Review?reportid=35236
07/22/20	Louisiana (Livingston)	1 dog	Asymptomatic	https://www.oie.int/wahis_2/public/wahid.php/Reviewreport/Review?reportid=35306
07/28/20	Texas (Brazos)	1 dog	Asymptomatic	https://www.oie.int/wahis_2/public/wahid.php/Reviewreport/Review?reportid=35408
07/29/20	Texas (Brazos)	1 cat	Asymptomatic	
08/04/20	North Carolina (Moore)	1 dog	Respiratory signs	https://www.oie.int/wahis_2/public/wahid.php/Reviewreport/Review?reportid=35408
08/10/20	Maryland (Hartford)	1 cat	Respiratory signs	https://www.oie.int/wahis_2/public/wahid.php/Reviewreport/Review?reportid=35525
08/11/20	Texas (Brazos)	1 dog	Nasal discharge	https://www.oie.int/wahis_2/public/wahid.php/Reviewreport/Review?reportid=35691
08/12/20	Texas (Brazos)	1 cat	Asymptomatic	
08/12/20	Texas (Somervell)	1 cat	Asymptomatic	
08/13/20	California (Contra Costa)	1 cat	Respiratory signs	https://www.oie.int/wahis_2/public/wahid.php/Reviewreport/Review?reportid=35525
08/17/20	Louisiana (Rapides)	1 cat	Respiratory signs	https://www.oie.int/wahis_2/public/wahid.php/Reviewreport/Review?reportid=35605
08/21/20	Texas (Brazos)	1 cat	Asymptomatic	https://www.oie.int/wahis_2/public/wahid.php/Reviewreport/Review?reportid=35691
08/21/20	Texas (Brazos)	1 dog	Asymptomatic	
09/06/20	Kentucky (Fayette)	1 cat	Respiratory signs	https://www.oie.int/wahis_2/public/wahid.php/Reviewreport/Review?page_refer=MapFullEventReport&reportid=35857

09/11/20	Texas (Brazos)	1 cat	Asymptomatic	https://www.oie.int/wahis_2/public/wahid.php/Reviewreport/Review?page_refer=MapFullEventReport&reportid=35946
09/14/20	Texas (Brazos)	1 dog	Respiratory signs	https://www.oie.int/wahis_2/public/wahid.php/Reviewreport/Review?page_refer=MapFullEventReport&reportid=35946
09/25/20	Alabama (Lee)	2 cats	Respiratory signs	https://www.oie.int/wahis_2/public/wahid.php/Reviewreport/Review?page_refer=MapFullEventReport&reportid=35973
10/01/20	Texas (Brazos)	1 dog	Digestive signs	https://www.oie.int/wahis_2/public/wahid.php/Reviewreport/Review?page_refer=MapFullEventReport&reportid=36309
10/02/20	Pennsylvania (Cumberland)	1 cat	Respiratory signs	https://www.oie.int/wahis_2/public/wahid.php/Reviewreport/Review?reportid=36309
10/22/20	Texas (Brazos)	2 cats	1 respiratory signs<br>1 asymptomatic	https://www.oie.int/wahis_2/public/wahid.php/Reviewreport/Review?page_refer=MapFullEventReport&reportid=37147
11/08/20	Florida (Okaloosa)	1 dog	Respiratory signs	
11/13/20	Pennsylvania (Washington)	1 dog	Respiratory signs	
11/15/20	Texas (Brazos)	1 cat	Asymptomatic	
11/17/20	Wisconsin (Eau Claire)	1 cat	Respiratory signs	
11/20/20	Texas (Tarrant)	1 cat	Vomiting and sneezing	
12/03/20	Kansas (Riley)	1 dog	Nasal discharge	
12/07/20	Virginia (Rockingham)	1 cat	Respiratory signs	https://www.oie.int/wahis_2/public/wahid.php/Reviewreport/Review?reportid=37493
12/14/20	Pennsylvania (Montgomery)	1 dog	Digestive signs	https://www.oie.int/wahis_2/public/wahid.php/Reviewreport/Review?reportid=37355
12/16/20	Florida (Polk)	1 cat	Respiratory signs	https://www.oie.int/wahis_2/public/wahid.php/Reviewreport/Review?reportid=37493
12/28/20	California (San Bernardino)	1 cat	Not available	https://www.oie.int/wahis_2/public/wahid.php/Reviewreport/Review?reportid=37602
01/01/21	Kansas (Pottawatomie)	1 cat	Digestive signs	
01/07/21	Arkansas (Saline)	1 cat	Nasal discharge	https://www.oie.int/wahis_2/public/wahid.php/Reviewreport/Review?reportid=37755
01/09/21	Tennessee (Knox)	1 cat	Respiratory signs	
**Mexico**				
08/14/20	Cuauhtémoc	1 dog	Respiratory signs	https://www.oie.int/wahis_2/public/wahid.php/Reviewreport/Review?page_refer=MapFullEventReport&reportid=37117
08/14/20	Iztacalco	1 dog	Respiratory signs	
11/13/20	Cuautitlán	1 dog	Respiratory signs	
11/23/20	Mztapalapa	2 dogs	Respiratory signs	
**SOUTH AMERICA**				
05/01/20	**Chili** (Santiago)	3 cats	Not available	https://www.oie.int/wahis_2/public/wahid.php/Reviewreport/Review?page_refer=MapFullEventReport&reportid=36236
10/05/20	**Brazil** (Cuiabá, Mato Grosso)	1 cat	Asymptomatic	https://www.oie.int/wahis_2/public/wahid.php/Reviewreport/Review?page_refer=MapFullEventReport&reportid=36324
**Argentina**				
09/01/20	La Plata, Buenos Aires	1 cat	Nasal discharge	https://www.oie.int/wahis_2/public/wahid.php/Reviewreport/Review?page_refer=MapFullEventReport&reportid=36560
10/09/20 Santiago	Del Estero	4 dogs	[5]	
		1 cat	weakening and anorexia	

[1] No information available on the mode of transmission; [2] Case linked to an outbreak on a mink farm; [3] Case in a neighborhood affected and cat authorized to leave; [4] At necropsy, a pituitary tumor was identified, which is thought to be the cause of the dog's neurological signs; [5] One of the dogs exhibits symptoms (conjunctivitis, cough, dyspnea, and weakening). The other three dogs were asymptomatic.
